# Severe leptospirosis complicated with multiorgan dysfunction successfully managed with plasma exchange: a case report

**DOI:** 10.1186/s13256-021-03135-3

**Published:** 2021-12-13

**Authors:** Manana Dewage Sankani Vishvara Kularathna, Senanayake Abeysinghe Mudiyanselage Kularatne, Manoji Pathirage, Pala Thanthirige Madhushi Anuradha Nanayakkara

**Affiliations:** Teaching Hospital Peradeniya, Peradeniya, Sri Lanka

**Keywords:** Leptospirosis, Zoonotic infection, Pulmonary hemorrhages, Myocarditis, Multiple organ failure, Plasma exchange

## Abstract

**Background:**

Leptospirosis is a common zoonotic infection caused by the spirochete *Leptospira*. The disease is more prevalent in the tropics, causing subclinical to severe illness leading to high morbidity and mortality.

**Case presentation:**

A 77-year-old healthy Sri Lankan man presented to the Teaching Hospital Peradeniya with severe leptospirosis complicated with acute kidney injury, pulmonary hemorrhages, myocarditis, and severe thrombocytopenia. He was deteriorating despite treatment with intravenous antibiotics and methylprednisolone boluses. He made a dramatic improvement with two cycles of plasma exchange.

**Conclusion:**

Therapeutic plasma exchange is a life-saving treatment modality in severe leptospirosis with multiorgan failure.

## Introduction

Leptospirosis is a zoonotic infection. The pathogen is harbored by both wild and domestic farm animals, including rodents, pigs, cattle, and dogs. The pathogenic genus *Leptospira interrogans* has more than 250 serovars arranged under 23 serogroups [1]. Transmission to humans occurs either by direct contact with infected animal tissue or body fluids, or indirectly from the environment contaminated with the pathogen entering via breaches of the skin or mucosal surface. In Sri Lanka, the farming community has a higher risk of contracting the infection, and even bathing in rivers and lakes, or casual exposure to wet soil carries the risk. After a short incubation period, the infection goes through septicemic and immune phases, causing the clinical manifestations. Historically, severe infection with jaundice was known as Weil’s disease. However, the clinical severity can vary from subclinical disease to severe multiorgan failure [2]. Thus, the clinical picture of such exotic infection could be similar to most situations of multiorgan dysfunction such as severe malaria, dengue, hantavirus infection, typhoid, rickettsial infections, immunosuppressives, sepsis, and radiation therapy. In Sri Lanka, there are about 3000–5000 suspected cases reported each year, with a case fatality rate of 1–2%, which is significant [3]. This report presents a case of severe leptospirosis successfully managed with plasma exchange. The effectiveness and efficacy of this treatment modality for severe leptospirosis are still under evaluation.

## Case presentation

A previously healthy 77-year-old Sri Lankan Tamil male from Galaha, a laborer at a vegetable farm, presented to the Teaching Hospital Peradeniya with a history of fever of 1-week duration. He was apparently well 1 week previously, and presented with high-grade fever along with arthralgia and myalgia. Apart from that, he had generalized headache with the onset of the illness. There were no associated respiratory symptoms, abdominal pain, vomiting, change in bowel habits, or urinary symptoms. He did not have significant past medical, surgical, or family history. He was unmarried and was an occasional alcohol consumer and a nonsmoker.

On examination, he was averagely built, conscious, ill looking, and severely dehydrated. He had cold peripheries. He was icteric, but there was no pallor or conjunctival suffusion. His pulse rate was 112 beats per minute, and his blood pressure was 77/48 mmHg. The abdominal, respiratory, and nervous system examination was unremarkable on admission.

**Figure:**
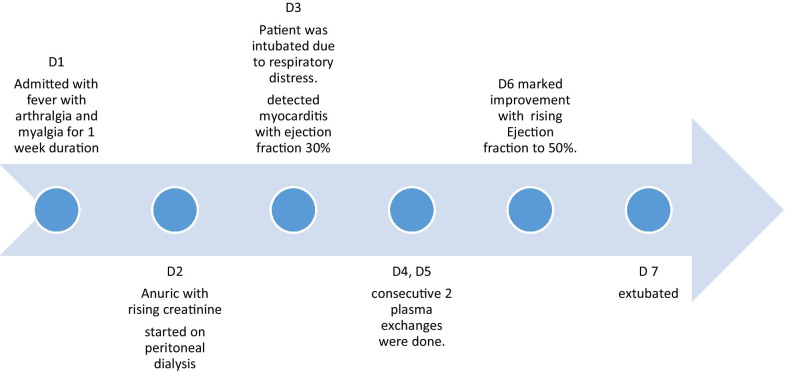

The patients’ complete blood count showed white blood cells (WBCs) of 4.46 × 10^9^/L (neutrophils 82%, lymphocytes 16%), platelet count 42 × 10^9^/L, hemoglobin (Hb) 13 g/dL, and hematocrit (HCT) of 37%. His C-reactive protein was 345 mg/L, and creatinine was 359 µmol/L. Serum bilirubin was 62.9 mmol/L with direct fraction of 42 mmol/L and indirect fraction of 21 mmol/L. Arterial blood gases showed pH 7.37, lactate 1.1 mmol/L, HCO_3_ 13 mEq/L, pCO_2_ 23 mmHg, pO_2_ 95 mmHg (Table 1).

**Table 1:** Investigations on admission and days later

Investigation	Value	Reference range
Complete blood count		
White blood cells	4.46 × 10^9^/L	3.4–9.6 × 10^9^/L
Neutrophils	82%	55–70%
Lymphocytes	16%	20–40%
Platelets	42 × 10^9^/L	150–450 × 10^9^/L
Hemoglobin	13 g/dL	13.2–16.6 g/dL
Hematocrit	37%	38.3–48.6%
C-reactive proteins		0–5 mg/L
Day 1	345 mg/L	
Day 7	19 mg/L	
Procalcitonin	37.94 ng/mL	< 0.15 ng/mL
Creatinine		59–104 µmol/L
Day 1	359 µmol/L	
Day 2	414 µmol/L	
Day 7	243 µmol/L	
Serum bilirubin	62.9 mmol/L	5.1–17 mmol/L
Direct	42 mmol/L	1.7–5.1 mmol/L
Indirect	21 mmol/L	3.4–12 mmol/L
Arterial blood gas		
pH	7.37	7.35–7.45
pO_2_	95 mmHg	75–100 mmHg
pCO_2_	23 mmHg	35–45 mmHg
HCO_3_^−^	13 mEq/L	22–26 mEq/L
Lactate	1.1 mmol/L	0.1–1 mmol/L
Electrocardiogram	Widespread T inversions	
2D echocardiogram		EF above 60%
Day 3	EF 30%, left ventricular global hypokinesia	
Day 6	EF 50%, improved left ventricular function	

A clinical diagnosis of leptospirosis was made, and the patient was started on intravenous C penicillin, intravenous ceftriaxone, and oral doxycycline. After adequate fluid resuscitation, the patient was started on intravenous noradrenaline infusion 0.6 mcg/kg/minute. On the following day, patient became anuric for 10 hours with rising creatinine of 414 µmol/L. Ultrasound scan of the kidneys showed acute renal parenchymal disease. The patient was started on peritoneal dialysis. On the third day of admission, the patient became dyspneic, and auscultation of the lungs revealed coarse and fine crepitations in the bilateral lung fields. The oxygen saturation dropped even with a continuous positive airway pressure (CPAP) mask, and was 81%. The chest X-ray showed bilateral patchy radio-opaque shadows. The patient was electively intubated, and intensive care was given. The patient was started on intravenous methylprednisolone 1 g daily for 3 days. The electrocardiogram (ECG) showed widespread T inversions, and the 2D echocardiogram showed ejection fraction (EF) of 30% with left ventricular global hypokinesia. His procalcitonin level was 37.94 ng/mL, and the platelet count was 54 × 10^9^/L. The patient was started on plasma exchange on the fourth day of admission, and 1840 mL plasma was removed along with transfusion of 1100 mL fresh frozen plasma and 1000 mL normal saline. Six units of platelets, intravenous tranexamic acid, and intravenous vitamin K were also started. The second plasma exchange was done on the fifth day, with removal of 1810 mL total plasma volume along with transfusion of 1500 mL fresh frozen plasma and 250 mL normal saline. His urine output markedly improved, and the peritoneal dialysis was withheld on the following day. Repeat 2D echocardiography showed improved left ventricular function, with ejection fraction of 50%. By the sixth day of admission, the patient’s inotrope requirement came down and we planned to wean off from the ventilator. Repeat chest x-ray showed clearing of opacifications in the lungs. The patient was extubated on the seventh day of admission. His serum creatinine came down to 243 µmol/L, and C-reactive protein (CRP) was 19 mg/L. Meanwhile, the diagnosis was confirmed as his serology showed positive enzyme-linked immunosorbent assay (ELISA) immunoglobulin M (IgM) for leptospirosis. By the tenth day of admission, the patient was discharged with complete recovery. In follow-up, the patient was reviewed after 2 weeks and found to be in good health.

**Figure:**
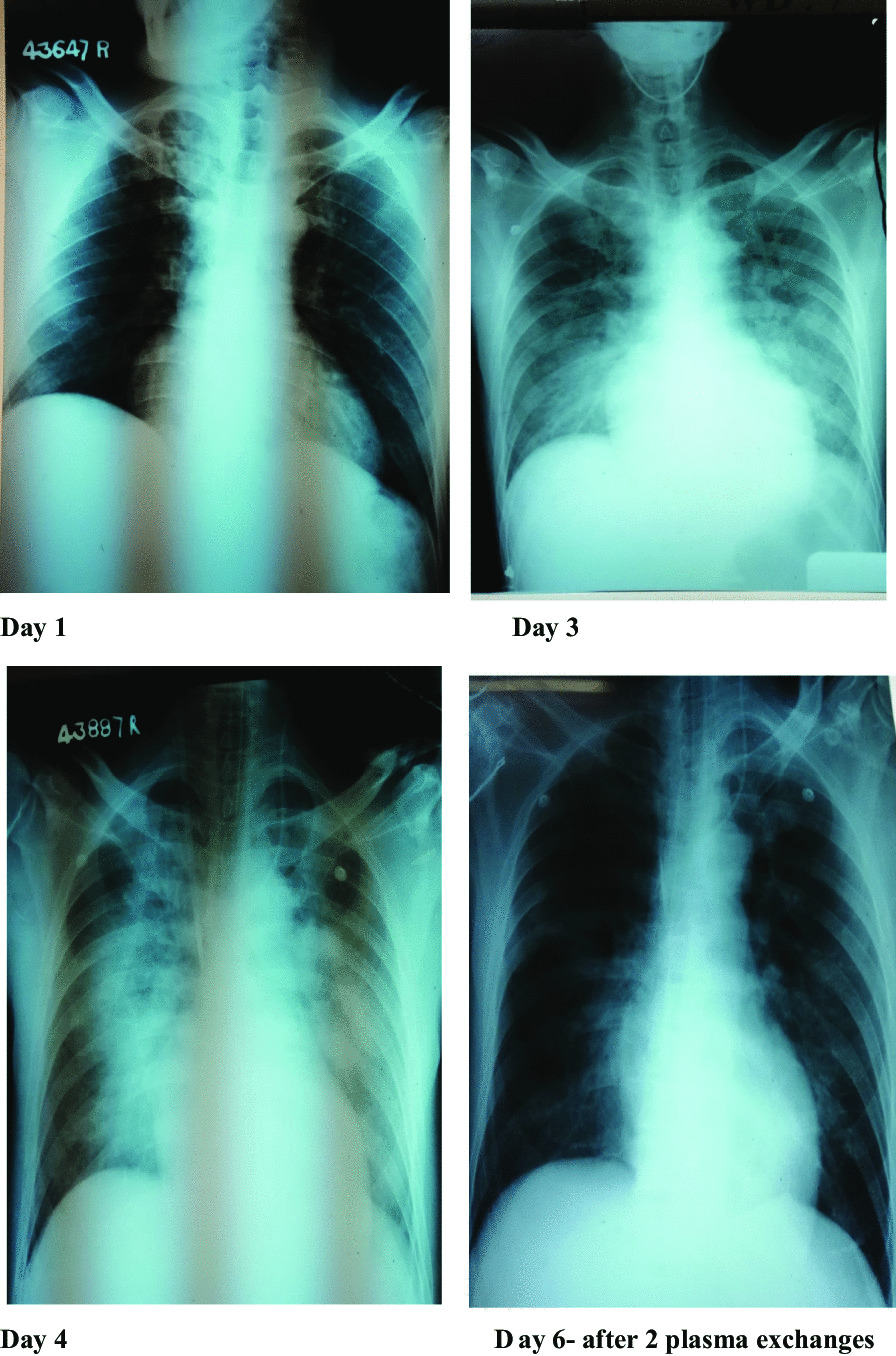

## Discussion

The patient was successfully managed, while deteriorating because of multiorgan failure, with the use of plasma exchange as last resort. The improvement was dramatic with increased EF of the left ventricle, increased urine output, and clearing of lung opacities. Leptospirosis is a commonly encountered zoonotic infection in Sri Lanka. Although many domestic and wild animals are recognized as reservoir hosts for *Leptospira*, among them, the brown rat (*Rattus norvegicus*) is recognized as the most important reservoir for human transmission. The pathogen remains dormant in the renal tubules of the reservoir animal and is shed via urine [4]. It is transmitted from animal to human through contact with infected animal urine on the mucous membranes or breaches of the skin [5]. The incubation period is 5–7 days on average [6]. Following penetration into the human body, there are two phases: the septicemic and immune phases [7]. During the septicemic phase, the patient has abrupt-onset fever, arthralgia, and myalgia, and *Leptospira* can be isolated from blood. Then, there will be a temporary settlement of symptoms before entering the immune phase. During the immune phase, there is a humoral response that causes clearance of the organism from most tissues [2]. During this phase, deposition of immune complexes will cause endothelial damage [2].

Our patient is a vegetable farmer and had a high risk of exposure to leptospirosis. Also, he presented with a clinical picture suggestive of leptospirosis and later became positive for ELISA IgM of leptospirosis, confirming the diagnosis according to World Health Organization (WHO) guidelines [6]. The patient had multiorgan failure with acute kidney injury, pulmonary hemorrhages, myocarditis, and hematological involvement with thrombocytopenia. Apart from routine usage of antibiotics, the patient was started on intravenous methylprednisolone 1 g daily for three consecutive days without success. After two cycles of plasma exchange on two consecutive days, the patient made a dramatic improvement from organ failure within only a few days.

Intravenous methylprednisolone is used to treat severe leptospirosis based on the immune-mediated pathogenesis of the disease, and it has been shown to be beneficial to reduce mortality [8]. The recommended dose used in Sri Lanka is intravenous methylprednisolone 1 g daily for 3 days. Early usage of methylprednisolone within 12 hours of onset of symptoms improved the outcome of the disease and reduced or delayed the need for mechanical ventilation [3]. Several case series and case reports have shown the benefit of steroid usage in severe leptospirosis. A Sri Lankan study of 227 patients in pre- and post-methylprednisolone periods demonstrated a significant reduction of death rates in the post-methylprednisolone group compared with the pre-methylprednisolone group (21.8% and 10.7%, respectively.) [8]. The benefit is more pronounced if given early in the multiorgan dysfunction [8]. A systemic review of usage of high-dose steroids in severe leptospirosis identified four studies with beneficial effects and one study that showed harmful effects leading to nosocomial infections [9].

Tissue damage will occur during the severe sepsis phase of leptospirosis because of systemic inflammation. Also, there will be tissue destruction due to immune complex-mediated mechanisms in the immune phase of the disease. Apart from that, with the usage of antibiotics, the release of an excessive amount of endotoxins by the organism’s death also contributes to the immune pathogenic process (Jarisch–Herxheimer reaction) [10]. Plasma exchange will minimize the tissue damage caused by the above mechanisms [11]. Also, there are beneficial effects by removing circulatory endotoxins, catabolic products, and inflammatory markers with the plasma exchange [12]. Additionally, high bilirubin levels occur in leptospirosis compared with the aminotransferase levels, as a result of impaired excretion of bilirubin caused by microcirculatory abnormalities and biliary obstruction [13]. High bilirubin levels in leptospirosis can cause toxic effects on the renal tubules leading to acute kidney injury. Therefore, removing bilirubin using plasma exchange has demonstrated rapid clinical response in severe leptospirosis [13].

The efficacy of plasma exchange was evaluated at the Teaching Hospital Karapitiya in Sri Lanka. A study of 53 patients with serologically confirmed complicated leptospirosis demonstrated a mortality rate of 36.4% in the therapeutic plasma exchange (TPE) only group, 21.4% in the intravenous immunoglobulin and therapeutic plasma exchange group, and 92.8% in the group that was given neither of these two therapies. Therefore, TPE showed a significant reduction of mortality in patients with severe leptospirosis complicated with multiorgan failure [14]. A few other case reports and studies demonstrated the beneficial effects of plasma exchange in severe leptospirosis. Trivedi *et al*. conducted a case series of 144 patients with serologically confirmed leptospirosis with mild pulmonary hemorrhages. Of them, 114 patients received two consecutive plasma exchanges followed by one dose of cyclophosphamide, while the rest were treated with symptomatic management. In the treatment group, 64.4% survived compared with the untreated group where the survival rate was 16.6% [5]. A 67-year-old patient with complicated leptospirosis was successfully treated with plasma exchange by Taylor *et al*. and demonstrated that plasma exchange plays an important role in recovery [11]. Kai-Chung Tse *et al*. applied plasma exchange to treat leptospirosis complicated with hyperbilirubinemia and renal failure and reported a rapid clinical recovery [13].

## Conclusion

We observed the survival benefit of therapeutic plasma exchange in multiple organ failure in severe leptospirosis. The plasma exchange would have reduced the systemic inflammation and the tissue damage caused by immune complex-mediated mechanisms in leptospirosis. More robust research studies are needed in the future to study the efficacy and effectiveness of plasma exchange in complicated leptospirosis.

## Data Availability

Data sharing is not applicable to this article as no datasets were generated or analyzed during the current study.

## References

[1] Dutta TK, Christopher M. Leptospirosis—an overview. 2005;53(June). 16121811

[2] Sheet F. Leptospirosis.

[3] Oludele J, Lesko B, Mahumane Gundane I, de Bruycker-Nogueira F, Muianga A, Ali S, Mula F, Chelene I, Falk KI, Barreto Dos Santos FGE. National guidelines on management of leptospirosis. Vol. 5; 2017. 10.4269/ajtmh.17-0317 PMC5817774 29016312

[4] Haake DA, Levett PN. Leptospirosis in humans. Curr Top Microbiol Immunol. 2015. 10.1007/978-3-662-45059-8_5. PMC4442676 25388133

[5] Trivedi SV, Vasava AH, Bhatia LC, Patel TC, Patel NK, Patel NT. Plasma exchange with immunosuppression in pulmonary alveolar haemorrhage due to leptospirosis. Indian J Med Res. 2010;131:429–33. 20418558

[6] WHO. Human leptospirosis: guidance for diagnosis, surveillance and control. Geneva: World Health Organization; 2003. p. iii.

[7] De Brito T, da Silva AMG, Abreu PAE. Pathology and pathogenesis of human leptospirosis: a commented review. Rev Inst Med Trop Sao Paulo. 2018. 10.1590/S1678-9946201860023. PMC5975557 29846473

[8] Kularatne SAM, Budagoda BDSS, De Alwis VKD, *et al*. High efficacy of bolus methylprednisolone in severe leptospirosis: a descriptive study in Sri Lanka. Postgrad Med J. 2011. 10.1136/pgmj.2009.092734. 21106802

[9] Soc TR, Med T, Rodrigo C, *et al*. High dose corticosteroids in severe leptospirosis: a systematic review. Trans R Soc Trop Med Hyg. 2014. 10.1093/trstmh/tru148. 25266477

[10] Bourquin V, Ponte B, Hirschel B, Pugin J, Martin P-Y, Saudan P. Severe leptospirosis with multiple organ failure successfully treated by plasma exchange and high-volume hemofiltration. Case Rep Nephrol. 2011. 10.1155/2011/817414. PMC3914130 24527237

[11] Reports C, Taylor D, Karamadoukis L. Plasma exchange in severe leptospirosis with multiorgan failure: a case report. J Med Case Rep. 2013;7(1):1. 10.1186/1752-1947-7-169. PMC3700770 23809518

[12] Cerdas-quesada C. Potential benefits of plasma exchange by apheresis on the treatment of severe icteric leptospirosis: case report and literature review q. Transfus Apheresis Sci. 2011;45:191–4. 10.1016/j.transci.2011.07.012. 21889407

[13] KaiChung T, PokSiu Y, KingMen H, *et al*. Potential benefit of plasma exchange in treatment of severe icteric leptospirosis complicated by acute renal failure. Clin Diagn Lab Immunol. 2002;9:482–4. 10.1128/CDLI.9.2.482-484.2002 PMC119963 11874897

[14] Fonseka CL, Lekamwasam S. Role of plasmapheresis and extracorporeal membrane oxygenation in the treatment of leptospirosis complicated with pulmonary hemorrhages. J Trop Med. 2018. 10.1155/2018/4520185. PMC6304550 30631369

